# Overexpression of LINC00551 promotes autophagy-dependent ferroptosis of lung adenocarcinoma via upregulating DDIT4 by sponging miR-4328

**DOI:** 10.7717/peerj.14180

**Published:** 2022-10-12

**Authors:** Xiong Peng, Rui Yang, Weilin Peng, Zhenyu Zhao, Guangxu Tu, Boxue He, Qidong Cai, Shuai Shi, Wei Yin, Fenglei Yu, Yongguang Tao, Xiang Wang

**Affiliations:** 1 Department of Thoracic Surgery, The Second Xiangya Hospital of Central South University, Changsha, Hunan, China; 2 Hunan Key Laboratory of Early Diagnosis and Precise Treatment of Lung Cancer, the Second Xiangya Hospital of Central South University, Changsha, Hunan, China; 3 Department of Pathology, School of Basic Medicine and Xiangya Hospital, Central South University, Changsha, Hunan, China; 4 NHC Key Laboratory of Carcinogenesis (Central South University), Cancer Research Institute and School of Basic Medicine, Central South University, Changsha, Hunan, China; 5 Department of Pathology, Xiangya Hospital, Key Laboratory of Carcinogenesis and Cancer Invasion, Ministry of Education, Central South University, Changsha, Hunan, China

**Keywords:** LINC00551, DDIT4, Ferroptosis, Autophagy, Lung adenocarcinoma

## Abstract

According to mounting evidence, long noncoding RNAs (lncRNAs) play a vital role in regulated cell death (RCD). A potential strategy for cancer therapy involves triggering ferroptosis, a novel form of RCD. Although it is thought to be an autophagy-dependent process, it is still unclear how the two processes interact. This study characterized a long intergenic noncoding RNA, LINC00551, expressed at a low level in lung adenocarcinoma (LUAD) and some other cancers. Overexpression of LINC00551 suppresses cell viability while promoting autophagy and RSL-3-induced ferroptosis in LUAD cells. LINC00551 acts as a competing endogenous RNA (ceRNA) and binds with miR-4328 which up-regulates the target DNA damage-inducible transcript 4 (DDIT4). DDIT4 inhibits the activity of mTOR, promotes LUAD autophagy, and then promotes the ferroptosis of LUAD cells in an autophagy-dependent manner. This study provided an insight into the molecular mechanism regulating ferroptosis and highlighted LINC00551 as a potential therapeutic target for LUAD.

## Introduction

Lung cancer is one of the most common malignant tumours worldwide, accounting for 2.2 million new cases and 1.8 million deaths in 2020, making it the leading cause of cancer-related mortalities globally (Sung et al., 2021). Lung adenocarcinoma (LUAD) is the most common histological subtype of non-small cell lung cancer (NSCLC). Despite the application of early screening and improvements in treatment, its prognosis is still dismal, as the overall five-year survival rate remains below 30% (Ettinger et al., 2021). Thus, it is urgently needed to improve the understanding of the molecular mechanisms underlying the pathogenesis of lung cancer and develop novel therapeutic approaches.

A recently identified regulated cell death (RCD) type caused by oxidative damage is known as ferroptosis. It is characterized by iron accumulation, lipid peroxidation, and subsequent plasma membrane rupture (Dixon et al., 2012). Emerging evidence has demonstrated that lncRNA plays a vital role in ferroptosis regulation (Mao et al., 2018). Though ferroptosis differs from other forms of RCD, some studies displayed that it is autophagy- or apoptosis-dependent cell death (Dixon et al., 2012; Yang et al., 2019). Recent studies reported that ferroptosis and autophagy are involved in the development and therapeutic responses of various cancers. They have both been suggested to have tremendous potential in cancer therapeutic strategies (Jiang, Stockwell & Conrad, 2021). However, the interactions between them are still largely unknown. The regulatory mechanism of ferroptosis in cancer and the cross-talk between ferroptosis and autophagy must be thoroughly investigated to develop a theoretical foundation for clinical therapy.

Accumulating evidence revealed that lncRNAs are essential for many cellular functions, including regulating gene expression and epigenetic signatures like cell viability, proliferation, and cell death (Huang et al., 2021). Our earlier research demonstrated the critical functions of lncRNAs by showing their participation in the regulation of malignant biological behaviours and RCDs of cancer (Mao et al., 2018; Wang et al., 2021). To the best of our knowledge, we were the first to evaluate the biological functions of LINC00551 in cancer (Peng et al., 2021). We performed RNA sequencing in oesophageal squamous cell cancer (ESCC) and identified that LINC00551 was expressed at a significantly low level in oesophageal cancer. It is down-regulation promoted cell proliferation and invasion in ESCC. We further analyzed its expression in other types of malignancies using The Cancer Genome Atlas (TCGA) datasets, and interestingly, LINC00551 was also remarkably expressed at a low level in LUAD, lung squamous cell carcinoma (LUSC), as well as in kidney malignancies such as kidney renal clear cell carcinoma (KIRC) and kidney renal papillary cell carcinoma (KIRP). Notably, KM-plotter analysis revealed that low LINC00551 expression is associated with a worse prognosis in LUAD and KIRC, indicating that it may act as a tumour suppressor gene.

In this study, we focussed on the function of LINC00551 in LUAD. Gain- and lost-function assays were performed to investigate the biological processes. We found that overexpression of LINC00551 suppressed cell viability while promoting the autophagy process and RSL-3-induced ferroptosis in LUAD cells. RNA sequencing results revealed that overexpression of LINC00551 up-regulated the mRNA level of DNA damage-inducible transcript 4 (DDIT4). As a competing endogenous RNA (ceRNA), LINC00551 binds to miR-4328 and up-regulates the target gene DDIT4. DDIT4 inhibits mTOR’s activity, promoting autophagy and ferroptosis of LUAD cells in an autophagy-dependent manner. In this study, we investigated the biological function of LINC00551 in LUAD and its molecular mechanism in ferroptosis regulation, which may provide a promising anticancer strategy that targets ferroptosis in LUAD.

## Material and Methods

### Cell culture

Human LUAD PC9 and A549 cells were obtained from the Chinese Academy of Science Cell Bank (Shanghai, China) and cultured in Roswell Park Memorial Institute 1640 medium (RPMI-1640; Gibco, Gaithersburg, MD, USA) supplemented with 10% fetal bovine serum (FBS; Gibco) in 5% CO_2_ at 37 °C. HEK-293T cells were maintained in a 10% FBS DMEM medium. RSL-3 and Ferr-1 were purchased from Selleck (TX, USA).

### Cell transfection

The plasmids carrying DDIT4 cDNA and shRNA were constructed in GeneChem (Shanghai, China). MiR-4328 Inhibitor was purchased from Rebobio (Guangzhou, China) and Mimics (miR-4328 mimics), and their controls (miR-NC) were synthesized by Sangon (Shanghai, China). All transfection procedures were performed using Lipofectamine 3000 (Invitrogen, Carlsbad, CA, USA). For further experiments, the cells were harvested for 48 h after transfection. Lentivirus infection resulted in the production of stable gene-expressing cells. These cells were infected by lentivirus for 72 h and treated with 2 µg/mL puromycin. The overexpression or shRNA efficiency was detected by quantitative real-time PCR (qRT-PCR). The sequences of mimics, inhibitors and shRNAs are listed in Table S2.

### Quantitative real-time PCR (qRT-PCR)

Total RNA was isolated from tissue or cell samples using Trizol reagent (Invitrogen, NY, USA). The RNA samples were reverse transcribed into cDNA using RT Master Mix (MCE, NJ, USA). The levels of targeted gene mRNA transcripts relative to the control were quantified in triplicate by qRT-PCR in StepOne System™ (Applied Biosystems, Waltham, MA, USA) using SYBR Green qPCR Master Mix with High ROX (MCE, NJ, USA) and specific primers. The data were analyzed using the 2^−ΔΔCt^ method. Primers used for qPCR were: LINC00551, 5′-CAGCCTTCAGTTGGAGGAAC-3′ and 5′-TGGCCAATGACGAATACTGA-3′; DDIT4, 5′-GATGCCTAGCCAGTTGGTAAG-3′ and 5′-CTAAACAGCCCCTGGATCTTG-3′; *β*-Actin, 5′-AAAGACCTGTACGCCAACAC-3′ and 5′-GTCATACTCCTGCTTGCTGAT-3′. This project was approved by the Ethical Committee on Scientific Research of the Second Xiangya Hospital of Central South University, Grant number 2020462.

### RNA sequencing

Three paired replicate RNA sequencing libraries were prepared from PC9 vector and PC9 LINC00551 overexpression cells. The six libraries were sequenced separately using the BGISEQ-500 sequencer, paired-end 150-bp read length, at 44 ×  10^6^ reads per sample. The statistical power of this experimental design, as calculated by RNApower (v 3.13), was 0.9. The clean reads were mapped to the reference genome using HISAT2 (v 2.0.4) (Kim, Langmead & Salzberg, 2015). Bowtie2 (v2.2.5) was applied to align the clean reads to the reference coding gene set, and then the expression level of the gene was determined by RSEM (v1.2.12) (Li & Dewey, 2011). The heatmap was drawn using the pheatmap package (v1.0.8), according to the gene expression in different samples. The differential expression analysis was performed using the DESeq2 (v1.4.5) with a Q value ≤ 0.05. Gene Set Enrichment Analysis (GSEA) was performed using the GSEA software. The sequence data was uploaded to NCBI GenBank with the following BioSample accessions: SAMN26553836, SAMN26553837, SAMN26553838, SAMN26553839, SAMN26553840, and SAMN26553841.

### Cell viability assays

Corresponding cells (3 ×10^3^ cells/well) were cultured in 96-well plates for 24 h, and the cell viability was assessed using the Cell Counting Kit-8 (CCK-8, Topscience, Shanghai, China), according to the manufacturer’s instructions. After incubating at 37 °C for 3 h, the absorbance was measured at 450 nm using the TECAN sunrise spectrophotometer (Tecan, Männedorf, Switzerland).

### Lipid reactive oxygen species (ROS) assays

The different A549 and PC9 cell groups were treated in triplicate with vehicle DMSO or RSL-3 (2.0 µM) for 48 h to determine the level of lipid ROS. The cells were then harvested and stained with 10 µM C11-BODIPY (581/591, ThermoFisher) for 30 min at 37 °C in the dark. After being washed, the cell samples were analyzed by a flow cytometer (BD, NJ, USA).

### Iron assay

The total iron and Fe^2+^ in the indicated cell samples were measured using an Iron Assay Kit (Sigma Aldrich, MO, USA). Briefly, ∼2 × 10^6^ cells were harvested and homogenized with four volumes of iron assay buffer, followed by centrifugation at 13,000 × g for 10 min at 4 °C. The supernatants were collected, and the differently diluted samples (95 µL each) were reacted with 5 µL of iron assay buffer or iron reducer for 30 min at room temperature in the dark to measure ferrous iron or total iron, respectively. Subsequently, the reacted samples in each well were mixed with a 100 µL iron probe and incubated for 60 min at room temperature in the dark. The absorbance of each well was measured at 593 nm using the TECAN sunrise spectrophotometer (Tecan, Männedorf, Switzerland). The total iron levels were quantified using the standard curves based on varying concentrations of standard iron provided. The levels of ferric iron were calculated by deducting ferrous iron from total iron.

### Western blot analysis

Cells were harvested, washed, and then lysed in the RIPA buffer (Beyotime, Shanghai, China) containing a cocktail of protease inhibitors (MCE, NJ, USA). After quantifying the protein concentration using a bicinchoninic acid (BCA) protein assay kit (Beyotime, Shanghai, China), the cell lysates (30 µg/lane) were separated by sodium dodecyl sulfate-polyacrylamide gel electrophoresis (SDS-PAGE) and transferred onto polyvinylidene fluoride (PVDF) membranes. The membranes were blocked in 10% non-fat dry milk in Tris-Buffered Saline and Tween-20 (TBST) for 1 h and incubated overnight with primary antibodies at 4 °C. After being washed three times. The bound antibodies were reacted with a secondary antibody. They were then visualized with a fluorescence imaging system (Sagecreation, Beijing, China) using enhanced chemiluminescence (Advansta, CA, USA). The primary antibodies included anti- *β*-Actin (20536-1-AP), anti-GPX4 (14432-1-AP), anti-p62 (18420-1-AP), anti-SLC7A11 (26864-1-AP), anti-DDIT4 (10086-1-AP), anti-LC3 (Proteintech, 14600-1-AP), and anti-ATG5 (Zen-bio, R23497).

### Transmission electron microscopy (TEM)

The different groups of cells were harvested, washed, and fixed in 2.5% glutaraldehyde and 2% paraformaldehyde (Merck, Darmstadt, Germany) and then post-fixed with 1% Osmium tetraoxide (OsO4). The cells were cut into ultrathin sections (70 nm), stained with uranyl acetate and lead citrate, and examined under a transmission electron microscope (Hitachi, Tokyo, Japan).

### Dual-luciferase reporter assays

A dual-luciferase reporter gene assay kit (#11402ES60; Yeasen, Shanghai, China) was used to perform the dual-luciferase assay. The 3′-UTR of DDIT4 wild type (DDIT4-WT) or DDIT4 mutant type (DDIT4-MUT) was cloned into the pGLO luciferase vector and transfected into 293T cells using Lipofectamine 3000 (Invitrogen, Carlsbad, CA, USA) with miR-NC or miR-4328 mimics. After 48 h, the luciferase activity of 293T was measured using a dual-luciferase reporter luminometer system (Promega Corporation, Madison, WI, USA).

### Xenograft lung tumour model in nude mice

The animal studies were approved by the Institutional Animal Care and Use Committee (IACUC) of Second Xiangya Hospital following the Guidelines of the Care and Use of Laboratory Animals issued by the Chinese Council on Animal Research. Briefly, female BALB/c nude mice at six weeks were obtained from Hunan SJA Laboratory Animal Co. Ltd. (Hunan, China) and kept in a specific pathogen-free environment. The mice were injected subcutaneously with 2 × 10^6^ indicated cells into the left or right flank for 21 days (PC9) or 28 days (A549) post-implantation. At the end of the experiment, the tumours were dissected and weighed.

### Statistical analyses

All statistical analyses were performed using SPSS 22.0 software, and graphs were made using the GraphPad Prism 8.0 software. The data were presented as mean ± SD. The student’s *t*-test analyzed the difference between the two groups. A paired *t*-test (for continuous data) or a chi-square test (for categorical data) were also used. The survival of animals was estimated by the Kaplan–Meier method and analyzed by the log-rank test. Ns represented non-significant (*p* >  0.05), while^∗^*p* < 0.05,^∗∗^*p* < 0.01, and^∗∗∗^*p* <  0.001 represented different degrees of significance.

## Results

### Overexpressing LINC00551 promotes the RSL-3-induced ferroptosis and autophagy in LUAD cells

**Figure 1:**
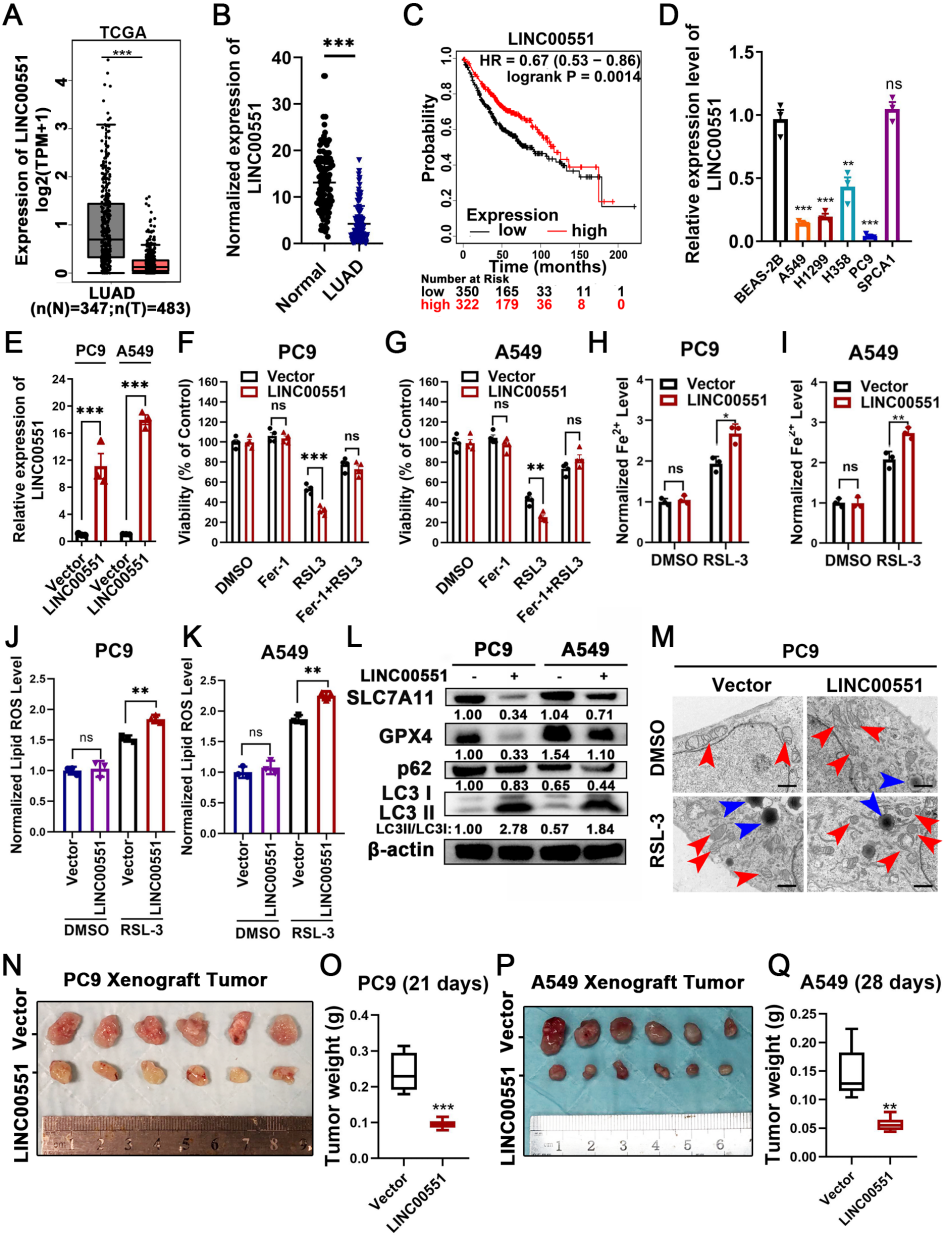
LINC00551 expression in LUAD and its effect on RSL-3-induced ferroptosis and autophagy in LUAD cells. (A) Relative LINC00551 expression (log_2_ (TPM+1)) transcripts in LUAD in TCGA datasets analyzed using GEPIA. (B) Quantitative analysis of LINC00551 transcripts in 62 pairs of LUAD and adjacent non-tumour tissues by qRT-PCR. (C) Overall survival of LUAD patients bearing high or low LINC00551 transcripts. (D) The relative levels of LINC00551 transcripts in normal lung epithelial cell line BEAS-2B and LUAD cell lines. (E) The relative levels of LINC00551 transcripts in the indicated PC9 and A549 cells by qRT-PCR. (F, G) The analysis of the viability of PC9 (F) and A549 (G) cells treated with ferrostatin-1 (2.0 µM) and RSL-3 (1.0 µM for A549, 2.0 µM for PC9). (H–K) The levels of ferrous iron (H, I) and lipid ROS (J, K) in the indicated PC9 and A549 cells. **L**. Western blot analyses of ferroptosis- and autophagy-related proteins in the indicated PC9 and A549 cells. (M) TEM characterization of mitochondria (red arrow) and lysosome (blue arrow) in the indicated PC9 cells. Scale bar = 1.0 µm. (N–Q) Xenograft tumours formed by PC9 (N) and A549 (P) cells transfected with LINC00551 expression vector or empty vector. Tumour weight of xenograft in LINC00551 overexpression and control group of PC9 (O) and A549 (Q) cells. ns: no significant (*p* > 0.05), ^∗^*p* < 0.05, ^∗∗^*p* < 0.01, ^∗∗∗^*p* < 0.001.

The level of LINC00551 transcripts in the TCGA was analyzed using the online tool GEPIA (http://gepia.cancer-pku.cn/index.html) (Tang et al., 2017). LINC00551 transcripts were significantly down-regulated in LUAD tissues compared to the non-tumour tissues (Fig. 1A). LINC00551 transcript levels were detected in 62 pairs of human clinical LUAD and adjacent normal tissues, and the results revealed that LINC00551 was poorly expressed in LUAD tissues (Fig. 1B). Stratification of 672 LUAD tissues with a cut-off value of median expression revealed that LUAD patients with lower levels of LINC00551 transcripts were associated with worse overall survival (OS) than those with higher levels of LINC00551 transcripts (Fig. 1C). LINC00551 generally displays lower expression in lung adenocarcinoma cell lines than normal lung epithelial cell line BEAS-2B (Fig. 1D). LINC00551 overexpressing PC9 and A549 cells were constructed to examine the impact of LINC00551 on ferroptosis (Fig. 1E). After being exposed to the ferroptosis inducer, RSL-3, the overexpression of LINC00551 significantly reduced the cell viability, but a ferroptosis inhibitor like Ferrostatin-1 (Fer-1) was able to restore this effect (Figs. 1F and 1G). In addition, LINC00551 over-expression significantly increased the concentrations of Fe^2+^ (Figs. 1H and 1I) and lipid ROS (Figs. 1J and 1K) in PC9 and A549 cells. Solute carrier family 7 member 11 (SLC7A11, also known as xCT) is a cystine/glutamate antiporter that imports extracellular cysteine for glutathione biosynthesis, ROS detoxification, and antioxidant activity. Glutathione peroxidase 4 (GPX4) is a lipid repair enzyme that represses ferroptosis by reducing lipid ROS. Both SLC7A11 and GPX4 are key repressors of ferroptosis. The decreased expression of SLC7A11 and GPX4 is generally considered a hallmark of ferroptosis. In this study, we found that overexpressing LINC00551 significantly reduced the protein expression levels of SLC7A11 and GPX4 (Fig. 1L), indicating that LINC00551 promotes ferroptosis in LUAD.

Recent evidence indicates that the molecular machinery of some selective autophagy (such as macroautophagy, a lysosome-mediated degradation process) facilitates ferroptosis (Hou et al., 2016). Excessive activation of autophagy seems to be an important driver of ferroptosis (Kang & Tang, 2017; Liu et al., 2020). The formation of autophagosomes is triggered by LC3 switching from LC3-I to -II (Kabeya et al., 2000). Whereas the autophagy substrate p62, an essential receptor for selective autophagy, functions by simultaneously interacting with LC3 (Gatica, Lahiri & Klionsky, 2018). Interestingly, RSL-3 exhibits broader and more vigorous activity in the upregulation of LC3-II or downregulation of p62 compared to other inducers (Li et al., 2021). We further detected the LC3II/I and p62 levels in RSL-3-induced cells. The results displayed that overexpression of LINC00551 significantly increased the conversion ratios of LC3-II to -I but decreased the relative levels of p62 expression in LINC00551 overexpressing PC9 and A549 cells (Fig. 1L). According to TEM analysis, RSL-3 treated LUAD cells depicted relatively smaller mitochondria with condensed membrane densities, a reduction of mitochondrial crista (red arrow), and an increase in the number of lysosomes (blue arrow) (Fig. 1M). Furthermore, tumor xenograft in nude mice exhibited a pronounced decline in size and weight of subcutaneous tumors in nude mice after overexpression of LINC00551 (Figs. 1N–1Q). These results indicated that the overexpression of LINC00551 promoted the RSL-3-induced ferroptosis and autophagy in LUAD cells.

### LINC00551 regulates DDIT4 expression by sponging miR-4328

**Figure 2:**
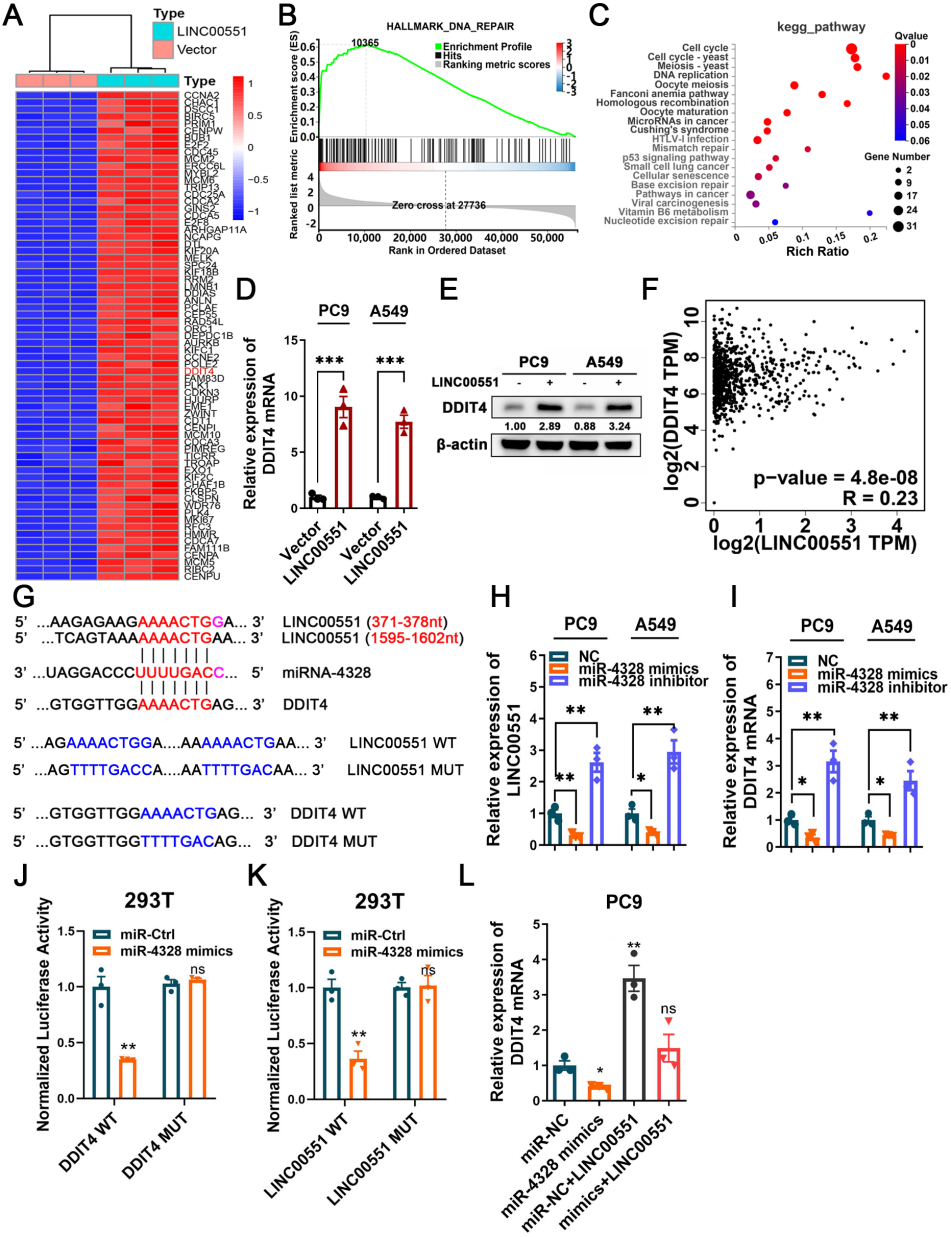
The regulatory mechanism of LINC00551 on DDIT4. (A) The heatmap of differentially expressed genes in LINC00551 over-expressing PC9 cell (fold change ≥ 6, *q*-value < 0.05) (B) GSEA for samples with high and low LINC00551 expression. (C) KEGG pathway analysis for biochemistry pathways studies in differential genes. (D) The relative levels of DDIT4 mRNA level in LINC00551 overexpressed cells by qRT-PCR. (E) The protein level of DDIT4 in LINC00551 overexpressed cells. (F) The correlation analysis between LINC00551 and DDIT4 mRNA levels in the TCGA database. (G) The predicted miRNA-4328 binding sequence in LINC00551 and DDIT4 and the generation of dual-luciferase reporter plasmids of wild-type (WT) or mutant (MUT). (H, I) RT-qPCR analysis of the relative levels of LINC00551 (H) and DDIT4 (I) transcripts in PC9 and A549 cells following transfection of miR-4328 mimics and inhibitors. (J, K) Luciferase reporter assay in 293T cells co-transfected with the wide type (WT) or mutated (MUT) DDIT4 (J) and LINC00551 (K) reporter vector and miR-4328 mimics. (L) The mRNA expression of DDIT4 mRNA in PC9 cells following ectopic expression of miR-4328 and/or LINC00551. ns: no significant (*p* > 0.05), ^∗^*p* < 0.05, ^∗∗^*p* < 0.01, ^∗∗∗^*p* < 0.001.

RNA sequencing was performed to investigate the potential molecular mechanism of LINC00551 in LUAD. The major genes up-regulated by LINC00551 are illustrated in Fig. 2A, most of which were primarily enriched in DNA repair and cell cycle, according to GSEA and KEGG analyses (Figs. 2B and 2C). DNA repair is an important part of the DNA damage response (DDR) network and is also one of the regulatory factors of cell cycle arrest (Li, Pearlman & Hsieh , 2016). Previously, we demonstrated that LINC00551 could regulate autophagy and ferroptosis in LUAD. Autophagy, a physiological self-eating process, is involved in DDR (Roshani-Asl et al., 2020). According to several recent investigations, it has been proven that DDR participates in ferroptosis (Chen, Tseng & Chi, 2020). DDIT4 is one of the main differentially expressed genes. It is a key regulator of rapamycin complex 1 (mTORC1) (Brugarolas et al., 2004), playing an important role in the regulation of DNA repair and RCDs, such as autophagy apoptosis and ferroptosis. RNA sequencing analysis revealed that the expression of DDIT4 mRNA was significantly increased in the LINC00551 overexpressing PC9 cells (Fig. 2A). Notably, the ferroptosis-related gene CHAC1 was increased in LINC00551 overexpressing PC9 cells (Fig. 2A). In LINC00551 overexpressing PC9 cells, the expression of DDIT4 was confirmed at both the mRNA and protein levels (Figs. 2D and 2E). Moreover, a positive correlation was found between LINC00551 and DDIT4 in the TCGA dataset (Fig. 2F). Bioinformatic analysis using Targetscan (http://www.targetscan.org/) and Starbase database (http://starbase.sysu.edu.cn/index.php) suggested that miR-4328 could bind to both LINC00551 and DDIT4 (Fig. 2G). Transfection with miR-4328 mimics significantly reduced the relative levels of DDIT4 mRNA and LINC00551 transcripts, while transfection with miR-4328 inhibitor significantly increased their levels (Figs. 2H and 2I). The dual-luciferase assay revealed that the luciferase activity of wild-type DDIT4 (DDIT4 WT) or wild-type LINC00551 (LINC00551 WT) was reduced by transfecting with miR-4328 mimics than the negative control group (miR-NC), but had no effect on the luciferase activities when mutated DDIT4 (DDIT4 MUT) or mutated LINC00551 (LINC00551 MUT) were transfected with it (Figs. 2J and 2K). PC9 cells co-transfected with miR-4328 mimics and LINC00551 partially restored the loss of DDIT4 gene in cells transfected with miR-4328 mimics only (Fig. 2L). These results indicated that miR-4328 can bind with both LINC00551 and DDIT4 and that LINC00551 regulates DDIT4 by acting as a ceRNA that competes with miR-4328 for binding.

### LINC00551 promotes autophagy and ferroptosis by regulating DDIT4

**Figure 3:**
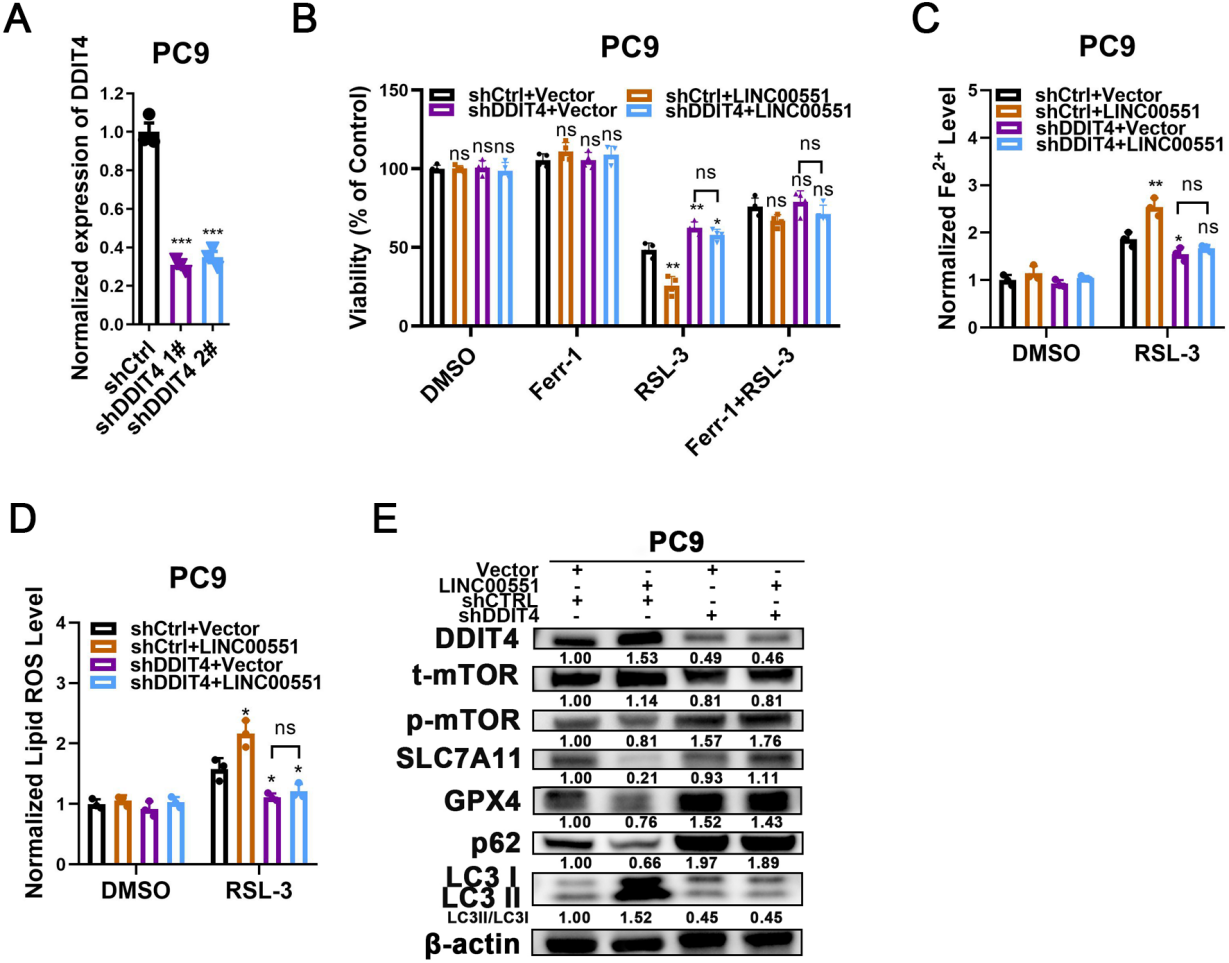
The role of DDIT4 in LINC00551-regulated autophagy and ferroptosis. (A) The relative levels of DDIT4 transcripts in the indicated PC9 cells. (B) The viability analysis of indicated PC9 cells treated with ferrostatin-1 (2.0 µM) and RSL-3 (2.0 µM). (C, D) The levels of ferrous iron (C) and lipid ROS (D) in the indicated PC9 cells following treatment with RSL-3 (2.0 µM). (E) Western blot analyses of the relative levels of ferroptosis- and autophagy-related proteins in the indicated PC9 cells. ns: no significant (*p* > 0.05), ^∗^*p* < 0.05, ^∗∗^*p* < 0.01, ^∗∗∗^*p* < 0.001.

To uncover the contributions of DDIT4 to LINC00551-induced effects, LINC00551 was transfected into PC9 cells with or without DDIT4 silencing (Fig. 3A). The results displayed that DDIT4 silencing significantly recovered the viability inhibition caused by LINC00551 overexpression (Fig. 3B). Moreover, DDIT4 silencing mitigated the levels of Fe^2+^ and lipid ROS caused by LINC00551 overexpression (Figs. 3C and 3D). As DDIT4 has been reported to inhibit mTOR activity (Brugarolas et al., 2004), the total/phospho(p)-mTOR was detected. The activity of mTOR was inhibited in LINC00551 overexpressed PC9 cells, and this effect was abrogated when DDIT4 was knocked down. Moreover, overexpression of LINC00551 remarkably increased the conversion ratios of LC3-II to -I but decreased the relative levels of SLC7A11, GPX4, and p62 expression in PC9 cells; these effects were abolished with the DDIT4 silencing (Fig. 3E). These data suggested that LINC00551 promotes RSL-3-induced autophagy and ferroptosis by regulating DDIT4.

### The LINC00551/DDIT4 axis regulates ferroptosis in an autophagy- dependent manner

**Figure 4:**
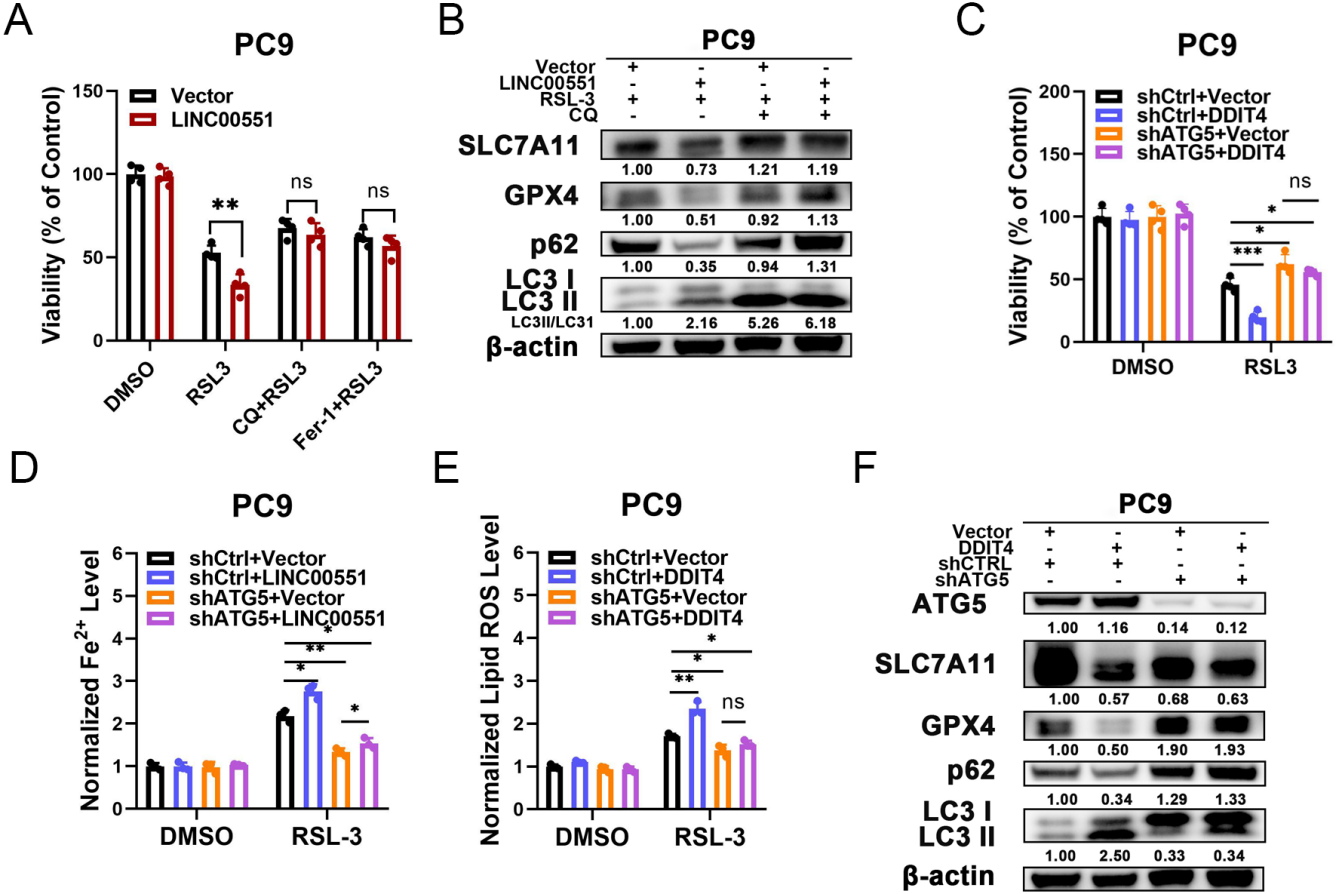
The relationship between autophagy and ferroptosis regulated by LINC00551/DDIT4 axis. (A) The viability of PC9 cells following treatment with RSL-3 (2.0 µM) in the absence or presence of chloroquine (20.0 µM) for 48 h. (B) Western blot analysis of the expression of autophagy and ferroptosis markers in the indicated PC9 cells following treatment with RSL-3 (2.0 µM) without and with chloroquine (20.0 µM) for 48 h. (C) The viability of PC9 cells following DDIT4 overexpression and/or ATG5 silencing. (D, E) The levels of ferrous iron (D) and lipid ROS (E) in the RSL-3 (1.0 µM, 24 h) induced PC9 cells following DDIT4 overexpression and/or ATG5 silencing. (F) Western blot analysis of the expression of autophagy and ferroptosis markers in A549 cells following treatment with RSL-3 in the absence or presence of ATG5 knockdown. ns: no significant (*p* > 0.05), ^∗^*p* < 0.05, ^∗∗^*p* < 0.01, ^∗∗∗^*p* < 0.001.

According to recent research, some types of autophagy may cause ferroptosis, and this process may be dependent on autophagy. However, the connections between the LINC00551/DDIT4 regulated autophagy and ferroptosis remain indistinct. Chloroquine (CQ), an autophagy inhibitor, or Ferr-1, can significantly reverse RSL-3-induced ferroptosis in LINC00551 over-expressed PC9 cells. This suggests that blocking autophagy diminishes the effect of LINC00551 over-expression on RSL3-induced ferroptosis in PC9 cells (Fig. 4A). Additionally, the treatment with CQ abrogated the effect of LINC00551 over-expression on autophagy and ferroptosis markers in PC9 cells (Fig. 4B). Meanwhile, the silencing of ATG5, a key regulator of autophagy, partially rescued the RSL-3-induced ferroptosis and eliminated the effect of DDIT4 overexpression on the RSL-3-induced ferroptosis in PC9 cells (Fig. 4C). ATG5 silencing attenuated the enhanced impact of DDIT4 overexpression on the RSL-3 increased concentrations of Fe^2+^ and lipid ROS in PC9 cells (Figs. 4D and 4E). Simultaneously, ATG5 silencing also abrogated the RSL-3-changed LC3-II formation and expression of p62, SLC7A11, and GPX4 in PC9 cells (Fig. 4F). These findings demonstrated that LINC00551/DDIT4 axis regulates the ferroptosis of LUAD cells in an autophagy-dependent manner.

## Discussion

Dysregulation of noncoding RNA expression and function occurs in different processes of human disease, including cancer. Both lncRNAs and miRNAs play vital roles in cancer. lncRNA can regulate its downstream mRNA by sponging miRNAs (Ransohoff, Wei & Khavari, 2018). Increasing evidence reveals that lncRNA is involved in ferroptosis regulation (Mao et al., 2018); however, the precise role of lncRNAs in ferroptosis remains elusive. In this study, we revealed that LINC00551 is expressed at a low level in LUAD tissues; it acts as a ceRNA to sponge miR-4328 and up-regulates DDIT4 expression, which inhibits the activity of mTOR and promotes autophagy; subsequently promotes RSL-3 induced ferroptosis in an autophagy-dependent way.

LINC00551 is a newly identified lncRNA. TCGA dataset analysis demonstrated that LINC00551 is significantly downregulated in ESCC, LUAD, and kidney malignancies like KIRC and KIRP. Low levels of LINC00551 were associated with poor prognosis in these cancers, indicating that LINC00551 plays a vital role in cancer development and progression. This study found that CHAC1, a ferroptosis-related gene, was significantly up-regulated in LINC00551 overexpressed PC9 cells. Taken together with our previous work, the heat shock protein beta-1 (HSPB1 aka HSP27) phosphorylation was controlled by the low expression of LINC00551 in ESCC (Peng et al., 2021). HSPB1 acts as a negative regulator of ferroptosis in several types of cancer. Thus, inhibition of HSPB1 expression and phosphorylation increases the anticancer activity of erastin-mediated ferroptosis (Sun et al., 2015). Hence, in this study, we focussed on the regulatory mechanism of LINC00551 in the ferroptosis of LUAD, and indeed, the results found that LINC00551 overexpression could promote RSL-3-induced ferroptosis in LUAD. However, the detailed molecular mechanism of ferroptosis regulation by LINC00551 remains unclear.

RNA sequencing analysis revealed that DDIT4 mRNA was significantly up-regulated in the LINC00551 overexpressing PC9 cells. DDIT4 is a key regulator of mTORC1 (Brugarolas et al., 2004), playing an important role in the regulation of DNA repair and RCDs, such as apoptosis and autophagy (Chen et al., 2016). DNA repair and autophagy play important roles in DDR (Roshani-Asl et al., 2020). Recent evidences suggest that DDR participates in ferroptosis (Chen, Tseng & Chi, 2020). Meanwhile, according to TCGA and GEO databases, a ferroptosis-related gene signature predicted DDIT4 as a ferroptosis driver (Tian et al., 2021). These evidences indicate that DDIT4 may be involved in regulating ferroptosis, but the underlying molecular mechanism remains indistinct. Our results revealed that LINC00551 overexpression promotes RSL-3 induced autophagy and ferroptosis and that DDIT4 silencing can rescue RSL-3 induced autophagy and ferroptosis in LINC00551 overexpressed PC9 cells, indicating that LINC00551 promotes the ferroptosis via DDIT4 in LUAD cells.

Ferroptosis is a new form of RCD, and triggering ferroptosis is a potential strategy for cancer therapy. Small molecular agents like sorafenib (Lachaier et al., 2014), sulfasalazine (Lewerenz et al., 2013), artemisinin along with its derivatives (Ooko et al., 2015), and nanomaterials (Kim et al., 2016) were all reported to be effective in inducing ferroptosis of cancer cells both *in vitro* and *in vivo*. Additionally, ferroptosis-based cancer therapies can be developed using gene technologies that either promote or suppress the expression of important genes, such as p53 and acyl-CoA synthetase long-chain family member 4 (ACSL4). Collectively, induction of ferroptosis might be a promising anticancer strategy. Thus, a better understanding of the molecular mechanisms underlying the regulation of ferroptosis can provide a scientific basis for developing therapeutic agents targeting ferroptosis in cancer.

Growing evidence reveals that different forms of RCDs are closely linked to each other (Campbell et al., 2018; Fitzwalter et al., 2018). However, little is known about regulating the connection between autophagy and ferroptosis. Autophagy is also critical in maintaining cell homeostasis under stress (White, Mehnert & Chan, 2015). Cell death may occasionally result from excessive or impaired autophagy (Liu & Levine, 2015). It is crucial for recycling ferritin and regulating the cell susceptibility to oxidative stress (Dowdle et al., 2014). Inhibition of NCOA4, a selective cargo receptor for ferritinophagy, inhibited ferritin degradation and suppressed ferroptosis. One of the key regulators of autophagy is the mTOR, a serine-threonine protein kinase known for suppressing autophagy by preventing the phosphorylation of ATG protein complexes. DDIT4 negatively regulates mTORC1 in response to cellular stressors (Kim & Guan, 2015). RSL-3 can block the mTOR activation to promote GPX4 protein degradation in pancreatic cancer cells (Liu et al., 2021), which facilitates the autophagy-dependent ferroptosis induced by RSL-3 (Chen et al., 2021). In this study, we found that LINC00551 overexpression up-regulated DDIT4, inhibited mTOR and facilitated autophagy, and further promoted ferroptosis in an autophagy-dependent manner.

The induction of ferroptosis has been considered a new strategy for anti-tumor therapies. LINC00551 could be a potential therapeutic target to induce ferroptosis for LUAD. However, lncRNA-based targeted cancer therapies are in their infancy. Modulating lncRNA transcription by altering the lncRNA-coded promoter activity is a promising approach to target lncRNAs for cancer therapy (Bhan, Soleimani & Mandal, 2017). Moreover, the inhibition of autophagy may diminish the anticancer activity of ferroptosis inducers (Song et al., 2018). The resistance to ferroptosis is a key barrier to tumor therapy. Combining ferroptosis activators with molecule-target agents for autophagy might become a novel therapeutic approach for anticancer therapy (Xu et al., 2020). DDIT4 promotes the autophagy-dependent ferroptosis of LUAD, acting as an mTOR inhibitor. Targeting DDIT4/mTOR signaling might be a new direction for LUAD therapy. However, proper animal models for testing and validation are urgently needed.

**Figure 5:**
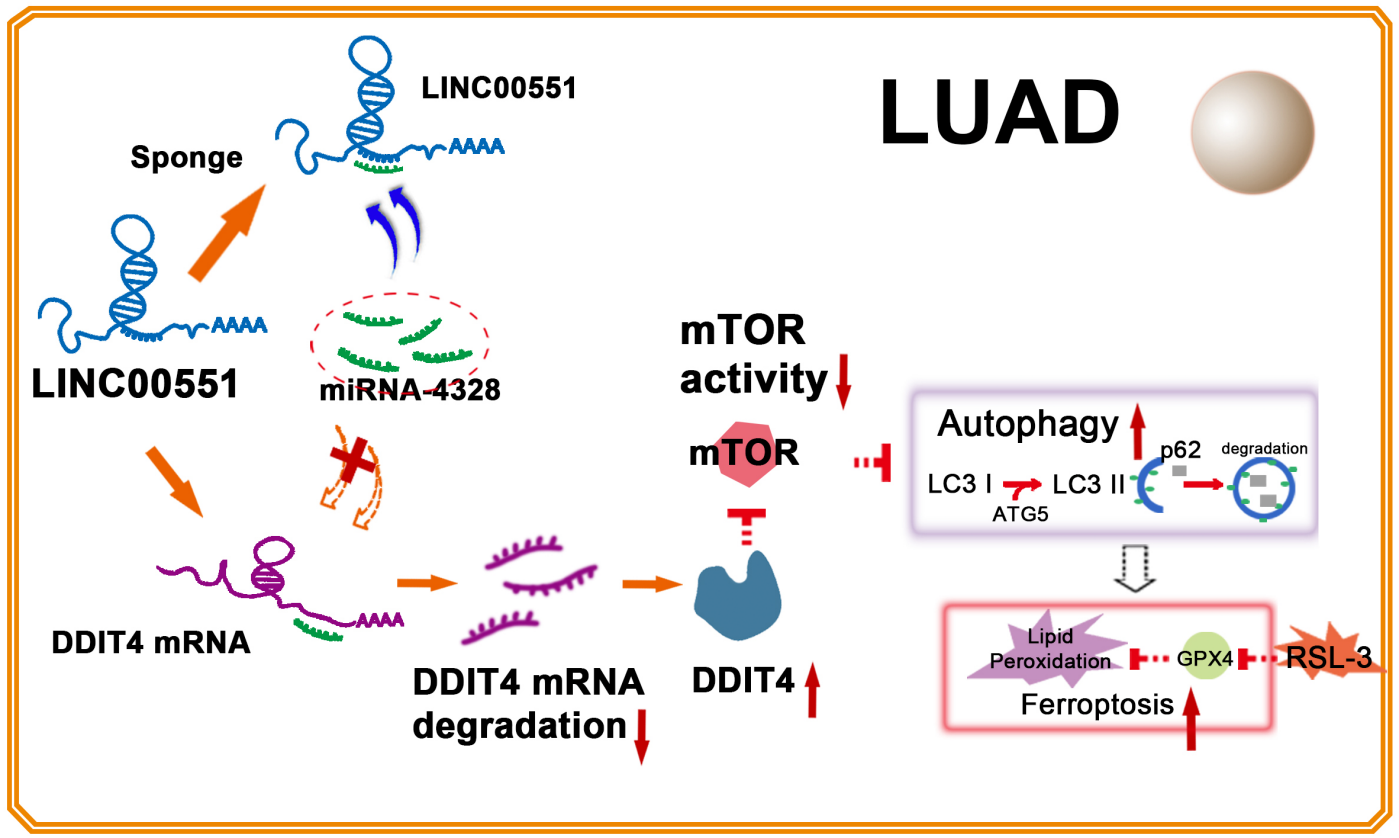
Illustration of LINC00551 action in LUAD. LINC00551 acts as a competing endogenous RNA and binds to miR-4328, subsequently up-regulating the target gene DDIT4. DDIT4 inhibits the activity of mTOR. DDIT4 inhibits the activity of mTOR, promotes LUAD autophagy, and then promotes the RSL-3 induced ferroptosis of LUAD cells in an autophagy-dependent manner.

## Conclusion

In conclusion, LINC00551 acts as a ceRNA that competitively binds to miR-4328, subsequently up-regulating the target gene DDIT4. DDIT4 inhibits the activity of mTOR, promotes autophagy, and RSL-3 induces ferroptosis of LUAD cells in an autophagy-dependent way (Fig. 5). Our study shed light on the molecular mechanism of ferroptosis regulation, laying the groundwork to develop therapeutic agents targeting the ferroptosis of LUAD and facilitating the advancement of targeted cancer therapies.

##  Supplemental Information

10.7717/peerj.14180/supp-1 Supplemental Information 1 Sequences for shRNAs,miRNA mimics and inhibitors

10.7717/peerj.14180/supp-2 Supplemental Information 2 Raw data of uncropped western blots

10.7717/peerj.14180/supp-3 Supplemental Information 3 Gene expression of RNA-seq in 3 pairs of LINC00551 overexpressing and control PC9 cells

10.7717/peerj.14180/supp-4 Supplemental Information 4 Raw data of figure 1HI 3C 4D, Fe2+

10.7717/peerj.14180/supp-5 Supplemental Information 5 Raw data of figure IJ, 3D, 4E, LIPID ROS

10.7717/peerj.14180/supp-6 Supplemental Information 6 Raw data of figure 1FG, 3B, 4AC, Cell viability

## Additional Information and Declarations

### DNA Deposition

The following information was supplied regarding the deposition of DNA sequences: The sequence data are available at GenBank: SAMN26553836, SAMN26553837, SAMN26553838, SAMN26553839, SAMN26553840, and SAMN26553841.

### Data Availability

The following information was supplied regarding data availability: The raw data for protein expression, cell viability, ROS, Fe2+ and the gene expression of RNA-seq are available in the Supplementary Files.
