# Supplementary figures and images for: Overexpression of LINC00551 promotes autophagy-dependent ferroptosis of lung adenocarcinoma via upregulating DDIT4 by sponging miR-4328
Source: PeerJ. 2022 Oct 12;10:e14180. doi: 10.7717/peerj.14180 (PMC9772902; doi:10.7717/peerj.14180)

Figure 1K

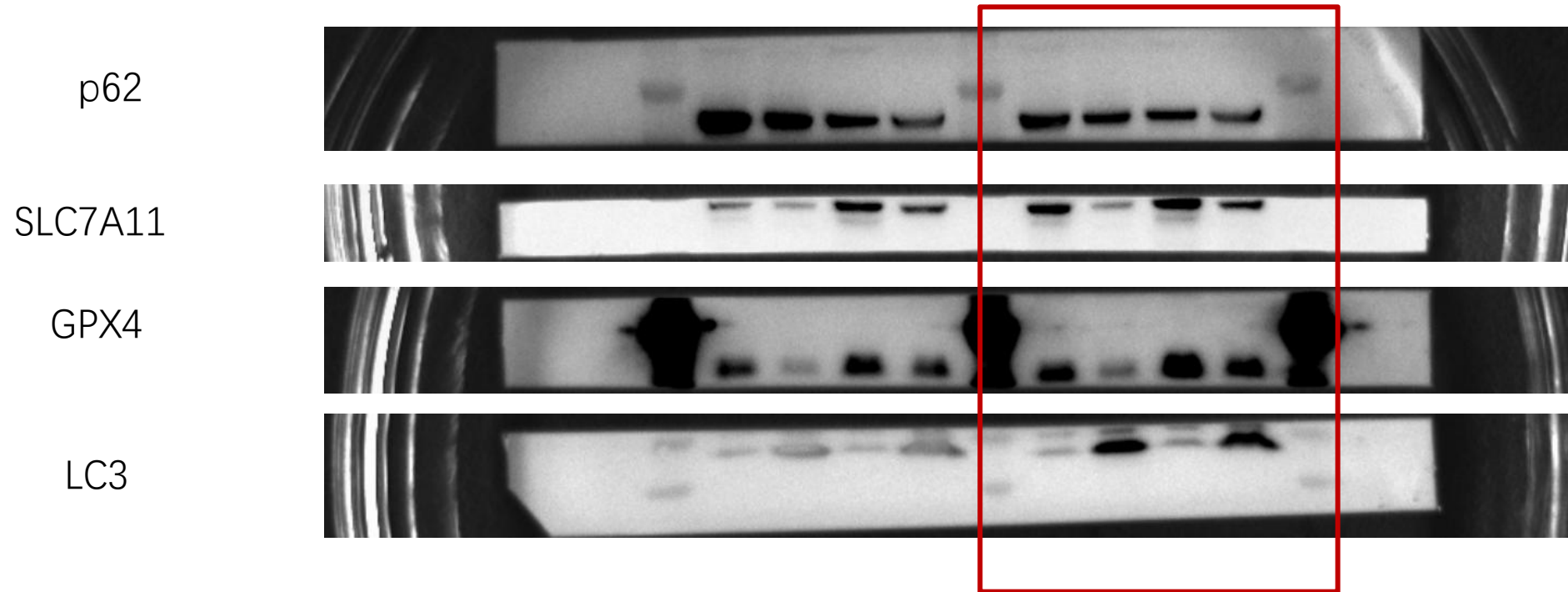

Figure 2E

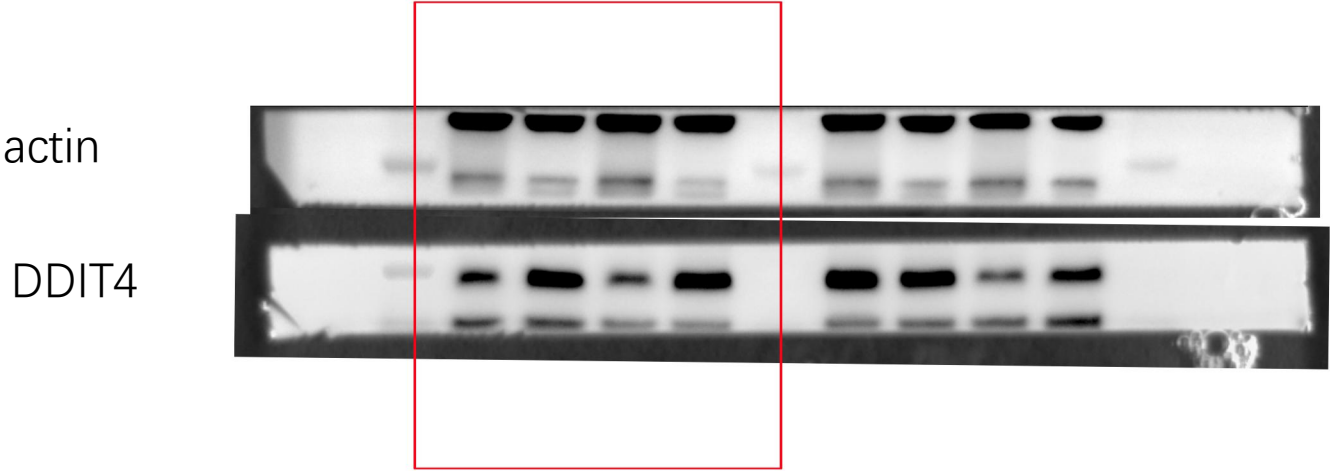

Figure 3E

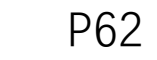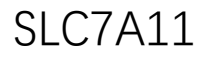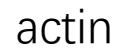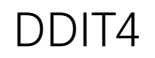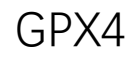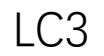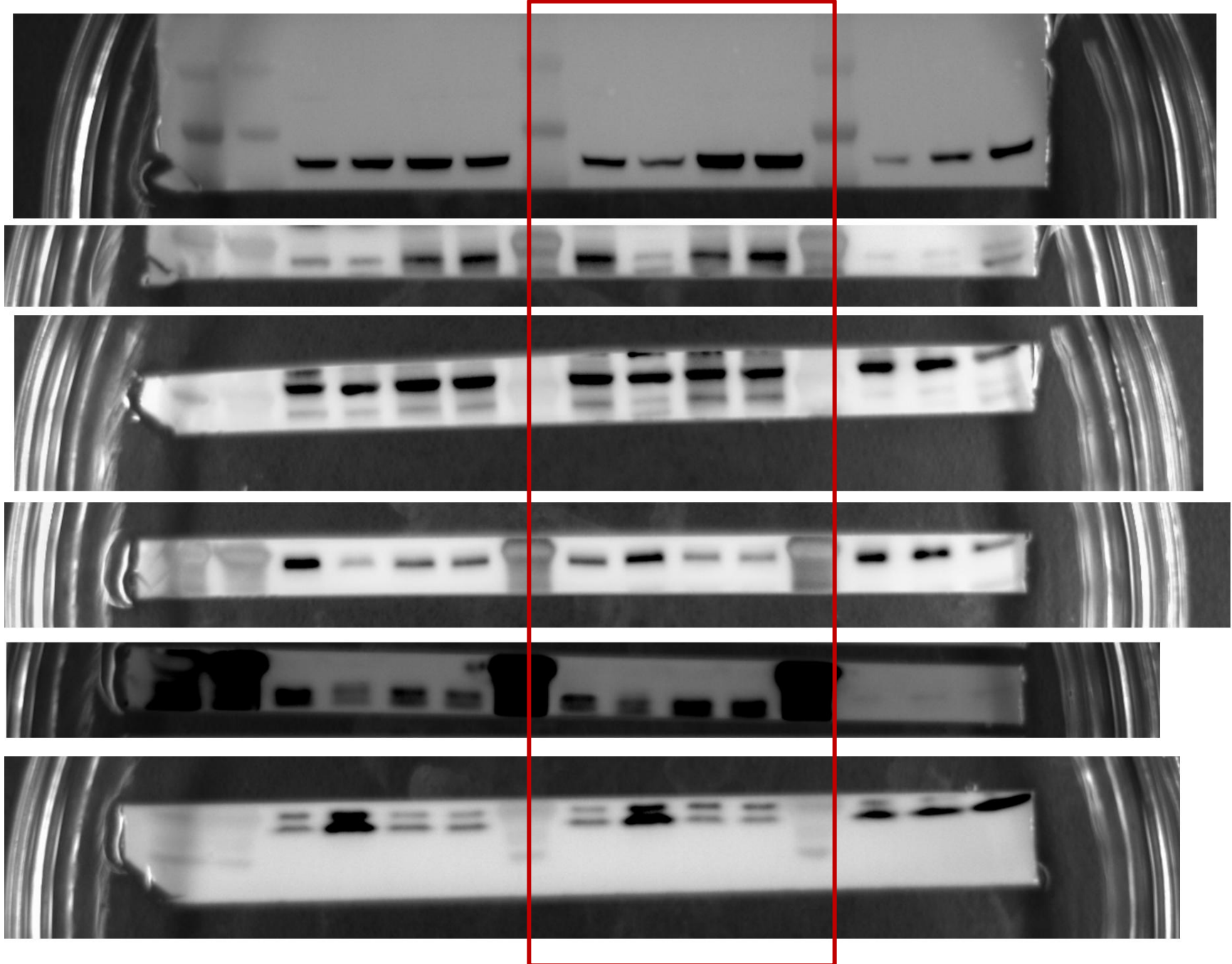

Figure 4B

P62

SLC7A11

actin

GPX4

LC3

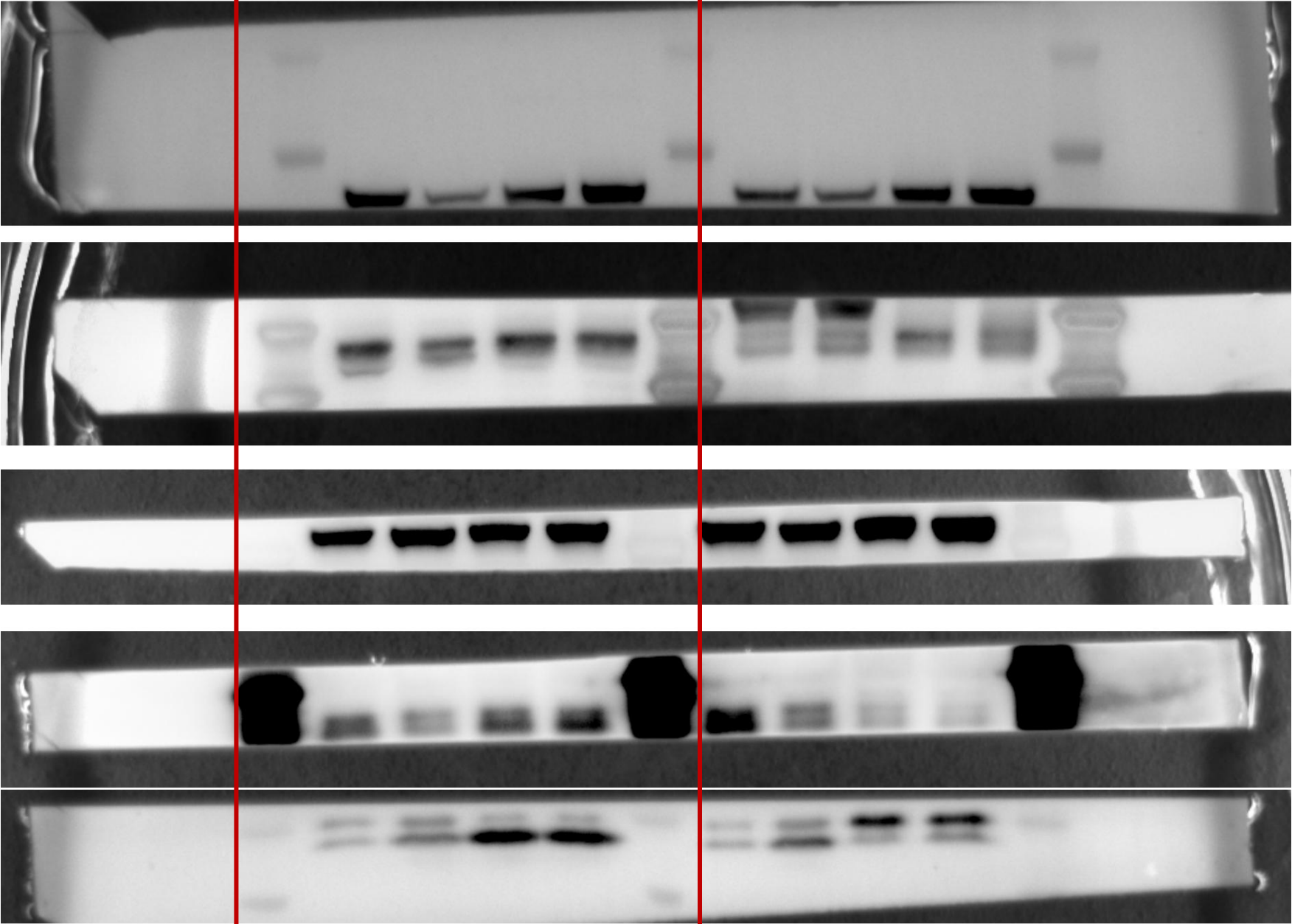

Figure 4F

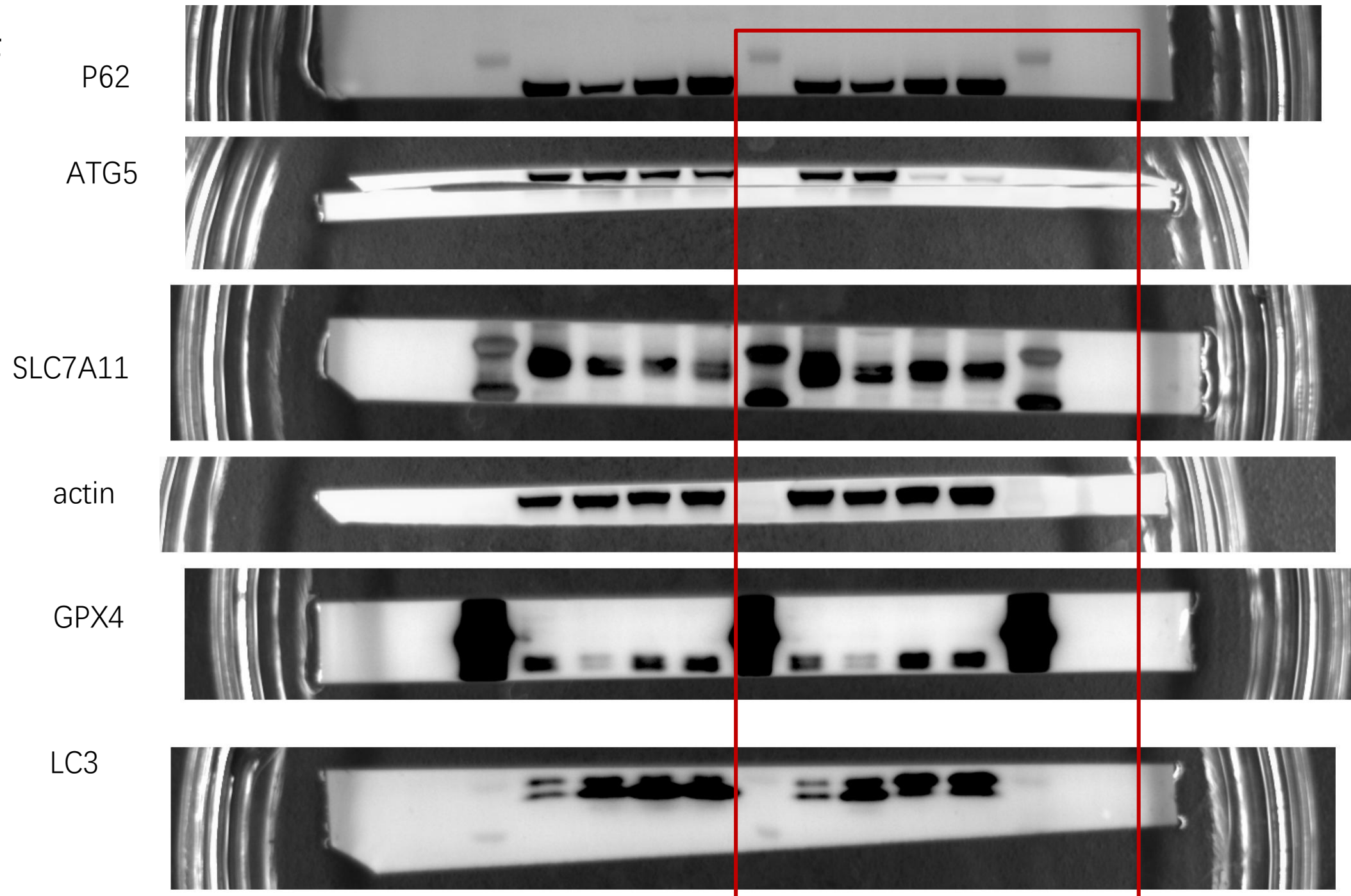

Supplement: Supplemental Information 2 [file peerj-10-14180-s002.pdf]
